# Long-term control with chemoradiation of initially metastatic mixed adenoneuroendocrine carcinoma of the rectum: a case report

**DOI:** 10.1186/s13256-019-1995-x

**Published:** 2019-03-23

**Authors:** S. Semrau, A. Agaimy, M. Pavel, D. Lubgan, D. Schmidt, A. Cavallaro, H. Golcher, R. Grützmann, R. Fietkau

**Affiliations:** 1 Department of Radiation Oncology, Friedrich-Alexander-Universität Erlangen-Nürnberg (FAU), Universitätsstraße 27, 91054 Erlangen, Germany; 2 Institute of Pathology, Friedrich-Alexander-Universität Erlangen-Nürnberg (FAU), Krankenhausstraße 8-10, 91054 Erlangen, Germany; 3 Department of Medicine, Division of Endocrinology, Friedrich-Alexander-Universität Erlangen-Nürnberg (FAU), Ulmenweg 18, 91054 Erlangen, Germany; 4 Clinic of Nuclear Medicine, Friedrich-Alexander-Universität Erlangen-Nürnberg (FAU), Ulmenweg 18, 91054 Erlangen, Germany; 5 Institute of Radiology, Friedrich-Alexander-Universität Erlangen-Nürnberg (FAU), Maximiliansplatz 1, 91054 Erlangen, Germany; 6 Department of Surgery, Friedrich-Alexander-Universität Erlangen-Nürnberg (FAU), Maximiliansplatz 1, 91054 Erlangen, Germany

**Keywords:** MANEC, Rectum, Chemoradiation, SBRT

## Abstract

**Background:**

Mixed adenoneuroendocrine carcinomas are highly malignant tumors with both adenocarcinomatous and neuroendocrine components. They can originate in any organ but are more common in the rectum. Due to their rarity, current treatment recommendations for mixed adenoneuroendocrine carcinoma are based on limited data and follow general guidelines for the management of adenocarcinomas and neuroendocrine neoplasms. Uncertainty regarding the efficacy of the available local and systemic treatment strategies is a compounding issue. Even those patients with locally limited disease have a relatively short life expectancy. In this report, we describe a case of deep rectal mixed adenoneuroendocrine carcinoma with long survival after chemoradiation.

**Case presentation:**

A 48-year-old Caucasian woman was diagnosed with a grade 3 rectal adenocarcinoma combined with a poorly differentiated large cell neuroendocrine carcinoma component and synchronous metastases (cT3cN1cM1) in both lobes of the liver in 2012. She received concomitant chemoradiotherapy followed by four additional cycles of cisplatin plus irinotecan. Initial treatment induced complete remission of the rectal tumor and liver metastases. Consequently, it was not necessary to surgically resect the primary tumor or any of the metastases. Three months after the end of treatment, one metastasis in the first segment of the liver showed regrowth, and stereotactic body radiotherapy of the metastasis and chemotherapy resulted in a clinical complete response. The patient has been recurrence-free for more than 5 years.

**Conclusions:**

Extended long-term control of a poorly differentiated metastatic (stage IV) mixed adenoneuroendocrine carcinoma is rare. The multimodal first- and second-line regimens of radiotherapy and chemotherapy described in this case report represent a new therapeutic approach. Encouraged by the results in this case, we compiled a review of the literature on mixed adenoneuroendocrine carcinoma.

## Background

Colorectal mixed adenoneuroendocrine carcinomas (MANECs) are highly malignant tumors [1]. Due to their rarity, only 1–2% of neuroendocrine tumors (NETs) of the large intestine are MANECs [2], and very few publications about these tumors exist. General treatment guidelines for these tumors with this mixed histology are lacking. According to the colorectal neuroendocrine carcinoma (NEC) guidelines issued by the European Neuroendocrine Tumor Society (ENETS), local treatment of metastatic MANEC is indicated in only a few cases, and metastatic MANEC has a poor prognosis [3]. The National Comprehensive Cancer Network guidelines for NETs, however, recommend surgical resection of metastases [4] based on a review of multiple NEC cases [5]. Surgery and radiotherapy are established modalities for the treatment of colorectal cancer and NETs in other locations, but scientific reports of explicit clinical experience validating their efficacy in the treatment of MANECs, especially metastatic stage MANECs, are still lacking.

In this report, we describe a case of a patient with metastatic MANEC treated by concurrent chemoradiotherapy (CRT) that resulted in long-term disease control. This case shows that local therapy, in particular CRT, should be considered in addition to sole polychemotherapy, even in poorly differentiated and metastatic MANEC, because of the curative potential.

## Case presentation

**Fig. 1:**
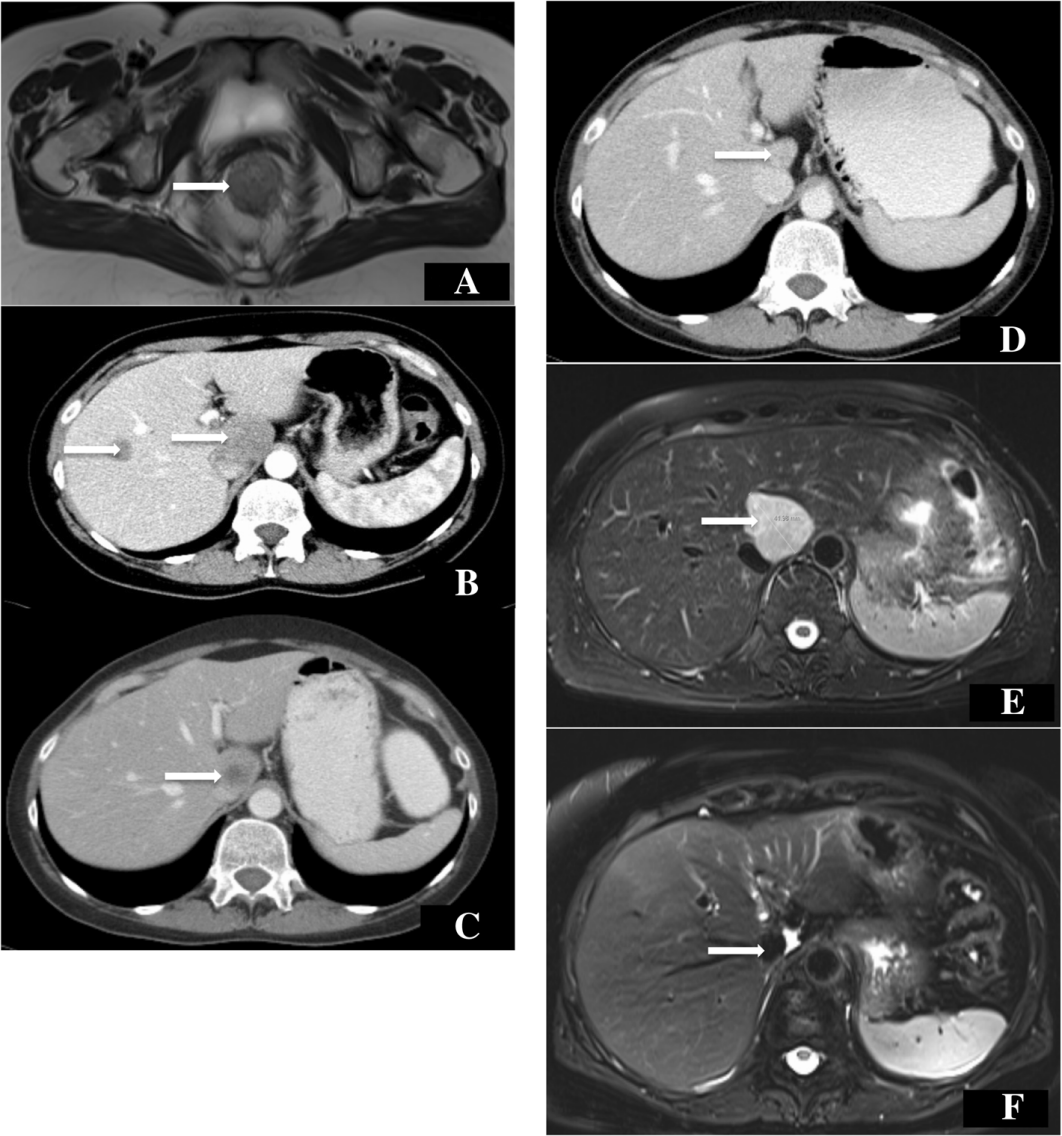
Deep seated, wall-penetrating rectal carcinoma (**a**, arrow, magnetic resonance imaging (MRI) with multiple bilateral liver metastases (**b**, arrow, computed tomography (CT) before treatment in April 2012. The patient reached partial remission after two cycles of cisplatin/irinotecan (**c**, residual metastasis in segment I with an arrow, CT) in June 2012 and complete remission after six cycles (**d**, arrow: no evidence of macroscopic metastasis in segment I, CT) in October 2012. View of segment I showing post-treatment recurrence in February 2013 (**e**, arrow, MRI) and status 5 years after stereotactic body radiotherapy and chemotherapy in February 2018 (**f**, no evidence of vital tumor: arrow, MRI)

**Fig. 2:**
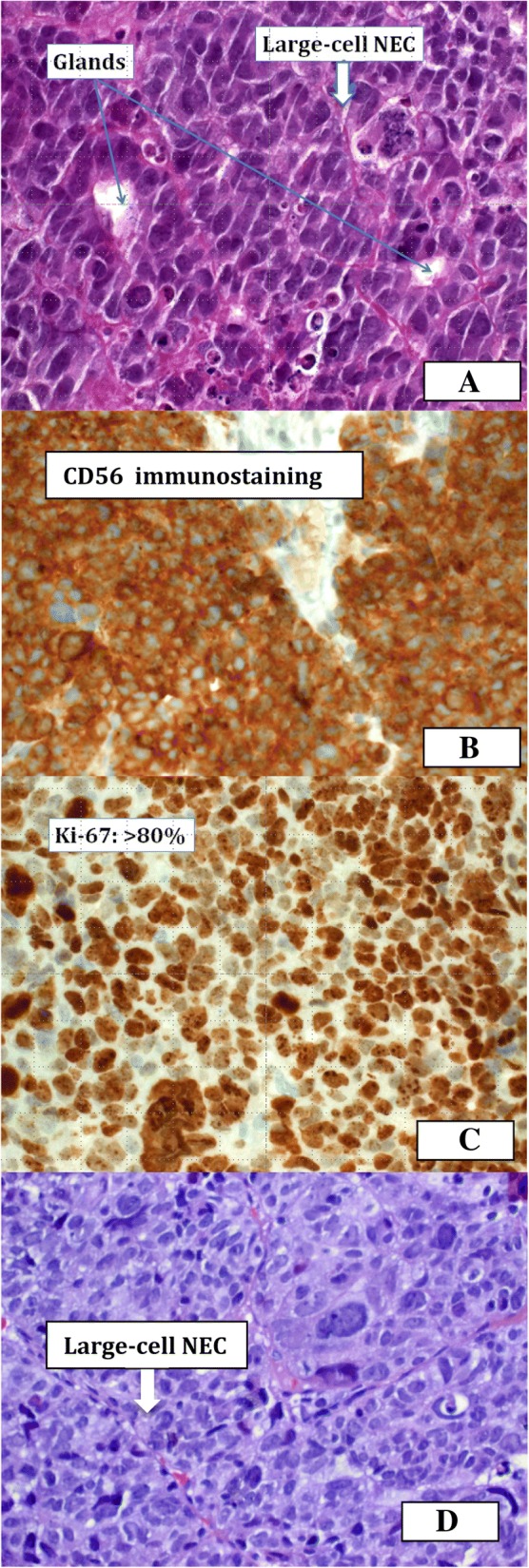
Findings of histological examination of the rectal tumor specimen. **a** Tumor with a small glandular component (hematoxylin and eosin stain (H&E stain)), adenocarcinoma (*thin arrow*), and a dominant neuroendocrine component (*thick arrow*). The neoplasm was CD56-positive (**b**) and had a high Ki67 proliferation index > 80% (**c**). An identical histologic pattern was seen in the liver metastasis (hematoxylin and eosin stain (H&E stain), **d**)

A 48-year-old Caucasian woman, Eastern Cooperative Oncology Group (ECOG) performance status 1, was diagnosed with locally advanced rectal carcinoma infiltrating the dental line with lymph node metastases. She was diagnosed by computed tomography (CT) (Fig. 1a) and proctoscopy (no image available) after presenting with problems with defecation, constipation, and tumor-related anemia (*see* Table 1 for treatment timeline). In fact, painful stenosis prevented endoscopic ultrasound. Significant preexisting diseases were not known, except hypothyroidism or any history of cancer in close family members. She had no occupational noxae. She did not smoke or drink substantial quantities of alcohol. Histological examination of a biopsy specimen of the tumor, which occupied the entire circumference of the rectum, revealed a poorly differentiated adenocarcinoma with a large cell NEC component (Fig. 2a) confirmed by strong diffuse staining for synaptophysin and CD56 (Fig. 2b) and comprising > 30% of the tumor in the biopsy material. The result of chromogranin A testing was negative. The patient’s Ki67 index was > 80%. Histology of the NEC component was consistent with grade 3 (G3) NEC of large cell type (Fig. 2c). More than ten metastases were also detected in both lobes of the liver by CT scan (Fig. 1b), so the patient’s TNM stage was cT3cN1cM1.

**Table 1:** Timeline

Diagnosis in 2012	Months 1–2	After 2 months	Months 3–6	After 6 months	Months 6–9	After 9 months	Months 11–12	Months 13–21	Month 23	Months 26, 29, 32, 35, 69
Diagnosis	Chemoradiation	Restaging	Chemotherapy	Restaging	No treatment	Staging	SBRT + chemotherapy	Chemotherapy	Restaging	Follow-up
MANEC of the rectum G3 cT3cN1cM1hep	Radiotherapy of the pelvic tumor + CDDP/IRI, two cycles	CT: Partial remission of the liver metastasis Endoscopy: Complete remission of the rectal tumor	CDDP/IRI, four cycles	CT: Complete remission of the liver metastasis of the rectal tumor		MRI: Recurrence of one liver metastasis	SBRT: Liver metastasis + one cycle CDDP/IRI	CDDP/IRI, eight cycles	MRI: Liver complete remission of the metastasis CT: Complete remission of the rectal tumor	MRI of liver and CT of thorax/abdomen: No recurrence

*Abbreviations: CDDP* Cisplatin, *CT* Computed tomography, *IRI* Irinotecan, *MANEC* Mixed adenoneuroendocrine carcinoma, *MRI* Magnetic resonance imaging, *SBRT* Stereotactic body radiotherapy

The patient received a regimen of cisplatin (CDDP; 20 mg/m^2^ on days 1–5, every 4 weeks) in combination with irinotecan (IRI; 50 mg/m^2^/day on days 1/8/15, every 4 weeks), an agent known for its efficacy in both colorectal cancer and NEC [6, 7]. In parallel, conventionally fractionated pelvic radiotherapy up to 50.4 Gy (reference point dose, intensity-modulated radiation therapy) was performed with the primary goal of alleviating pain and preventing obstruction by achieving maximum response. Initially, the patient received a red blood cell transfusion and sodium picosulfate against constipation. For antiemetic prophylaxis during all chemotherapy cycles, she received aprepitant (125 mg/day, d1; 80 mg/day, d2–5), ondansetron (16 mg/day), dexamethasone (12 mg/day, d1; 8 mg/day, d2–5), and pantoprazole 40/mg/day and enoxaparin sodium 40 mg/day.

At the end of chemoradiation, the patient experienced rectal pain, which was treated with tramadol (3 × 100 mg/day), and fatigue. Parenteral nutrition was required because of diarrhea (Common toxicity Criteria for Adverse Events version 5.0 [CTC] grade III) and dehydration (CTC grade III). The patient had port-related sepsis (*Staphylococcus epidermidis* in blood culture), which was successfully treated with vancomycin (2 × 1 g/day, intravenous), and a urinary tract infection (*Escherichia coli*), which was treated with ciprofloxacin (2 × 400 mg/day, intravenous). She needed red cell blood transfusions for anemia during the first cycle (CTC grade III) (*see* Table 2) and filgrastim 480μg/0.5 ml for 6 days for the treatment of leukopenia (CTC grade IV) at the end of the second cycle of chemotherapy. There were no unexpected events or clinical examination results. A summary of relevant laboratory parameters at baseline and during treatment is provided in Table 2.

**Table 2:** Eastern Cooperative Oncology Group Performance Status and laboratory data at diagnosis, during treatment, two month after treatment and last follow up at 69 month

Diagnosis in 2012		Month 1–2	after 2 month	Month 3–6	after 6 month	Month 6–9	after 9 month	Month 11 + 12	Month 13–21	Month 23	Month 69
ECOG Performance Status											
	1	2	2	1	1	0	0	0	2	0	0
Laboratory Data at the start of treatment, during the treatment as maximum toxicity, restaging and last follow											
White blood cell (×10^3^ /μl)	7.5	0.6	3.8	1.4	2.2	3.6	4.6	6.6	1.5	6.6	8.7
Hemoglobin (g/dl)	8.8	7.2	10.0	8.4	8.2	10.9	13.3	10.0	8.8	11.9	10.6
Red blood cell (× 10^6^ /μl)	4.3	2.6	3.4	2.7	2.6	3.1	4.1	3.0	2.5	3.6	4.1
Platelet (×10^3^ /μl)	431	74	144	178	240	225	289	209	113	245	491
Creatinine (mg/dl)	0.83	1.42	1.48	1.33	0.85	1.00	0.77	1.09	1.03	1.08	0.98
AST (U/l)	214	52	21	79	32	43	26	36	37	25	16
ALT (U/l)	129	117	16	118	30	50	22	41	53	18	17
Total Bili (mg/dl)	0.8	0.7	0.3	0.9	0.4	0.5	0.4	0.7	0.5	0.3	0.4
GGT (U/l)	50	176	62	70	31	37	24	91	123	36	16
Albumin (g/l)	34.4	22.7	30.9	30.9	40.7	34.9	45.9	33.9	29.3	42.1	38.5
INR	1.0	0.99	1.01	1.01	1.03	1.03	–	0.98	0.99	–	0.95

As the CT examination performed immediately after the end of radiotherapy showed only partial remission of the liver metastases (Fig. 1c), four additional cycles of modified CDDP/IRI (CDDP 20 mg/m^2^ on days 1–4, every 4 weeks; IRI 50 mg/m^2^/day on days 1/8/15, every 4 weeks) with prophylactic treatment mentioned above were administered after the end of chemoradiotherapy. No toxicity CTC grade III or IV was observed, but the patient had temporary need of a fentanyl patch for rectal pain treatment. Ultimately, she had ECOG I with no pathologic findings in the physical and neurological examinations.

In light of clinical complete remission of the deep rectal cancer and improvement of rectal stenosis, confirmed by simple proctoscopy with direct visualization, surgical resection was not performed, owing to uncertainty regarding the chances of preserving fecal continence. Complete remission of the liver metastases seen in the CT scan was also achieved after a total of six cycles of CDDP/IRI (Fig. 1d).

Recurrence of an initial metastasis in segment I was detected after a treatment-free interval of 3 months (Fig. 1e). Examination of a liver biopsy specimen revealed poorly differentiated NEC (Fig. 2d). The patient underwent eight new cycles of CDDP/IRI (CDDP 20 mg/m^2^ d1–3; IRI 60 mg/m^2^ d1, d8, d15; cycles IV to VIII with 60% of the dose) with the same prophylactic treatment and stereotactic body radiotherapy of the liver metastasis within the first cycle of chemotherapy. The fractionation scheme was 15 × 3 Gy (reference point dose), 60 Gy (equivalent dose in 2-Gy fractions with α/β = 10). During this treatment, there was a port infection (CTC grade III, *S. epidermidis*) treated with vancomycin (2 × 1 g/day, intravenous), but no other higher-grade toxicity or relevant neurologic or physical findings during hospital stay or outpatient visits, which took place at least once per week.

**Fig. 3:**
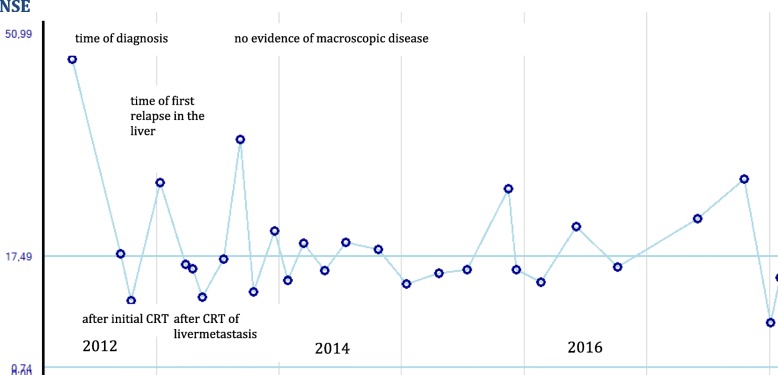
Neuron-specific enolase (NSE) tests showed peak levels before treatment and at the time of metastasis recurrence. Low levels were reached after first chemoradiation (CRT) at the time of complete response (CR) and after second chemoradiation of the liver metastasis

Treatment resulted in complete remission of the metastasis (Fig. 1f). Serum neuron-specific enolase, an independent marker of overall survival of NETs (upper limit of normal, 17.49 ng/ml), also decreased in parallel with the treatment cycles (Fig. 3).

The patient was followed up by CT scan of the chest and abdomen, as well as MRI of the liver every 6 months, and was tumor-free and symptom-free for 5 years and had no signs of impaired liver function or late toxicity after rectal radiotherapy. Results of all clinical and laboratory investigations remained unremarkable (Table 2). The patient’s last follow-up examination was in the autumn of 2018.

## Discussion

Our patient’s case demonstrates that excellent long-term results can be achieved by using a combination of local and systemic therapy in first- and second-line treatment of MANECs, even in cases with multiple synchronous visceral metastases of the NEC component, when success is measured in terms of organ preservation and systemic disease control over several years. To the best of our knowledge, this is the first time that such a successful long-term outcome has been reported in the literature. Due to the rarity of the disease, this has implications for treatment planning in similar cases.

Such information is particularly important because, although rare, NETs and carcinomas of the rectum are being diagnosed in increasing numbers. Their incidence has been steadily rising for decades; for instance, from 0.1 per 1,000,000 persons in 1973 to 1.0 per 100,000 in 2014 [8]. Although the percentage of MANECs within this group of tumors is low, their incidence can be expected to rise as awareness of this tumor entity increases.

MANECs represent an aggressive subgroup in the spectrum of mixed neuroendocrine-nonneuroendocrine neoplasms. According to the current World Health Organization classification of neuroendocrine neoplasms, both tumor components must make up at least 30% of the tumor [9]. The MANEC components may present two distinctive separated components within a single gross mass (collision tumor-like), blend with each other (composite tumors), or (less commonly) show dual (amphicrine-type) differentiation within the same cells. Diagnosis based on biopsy specimens might be impossible if only a single component is represented. Although both components can/should be graded, the neuroendocrine component is usually prognostically limiting, and it corresponds to poorly differentiated NEC of either small cell type (less frequently) or large cell type; our patient had the latter type [1]. The large and small cell pattern of the NEC in MANEC corresponds to the most frequent pulmonary counterparts. Very rarely, the neuroendocrine component may display a well-differentiated morphology (NET morphology) and should be graded on the basis of Ki67 index similar to pure NETs of the gastroenteropancreatic system into NET G1 (Ki67 < 3%), NET G2 (Ki67 3–20%), or rarely NET G3 (Ki67 > 20%). Although Ki67 is usually very high in NEC (from > 50% to 100%), their classification is mainly morphology-based (poor cytological and architectural differentiation) and not on the basis of their high Ki67 index.

The prognostic relevance of the different tumor types and grades is not clear yet. In this sense, MANECs differ from other pure NETs. Especially if in the metastatic stages, patients with MANEC generally have very poor survival, regardless of whether the neuroendocrine component has a low or high proliferative index [10].

MRI for the diagnosis of lymph node metastasis is presumably less sensitive for MANECs than for pure adenocarcinomas [11].

As no study- or guideline-based general treatment recommendations for mixed NECs exist, clinicians look to recommendations for colorectal cancer and NEC for guidance. This has led to critical discussions regarding the necessary extent of surgery, the need for radiotherapy, and the type of chemotherapy.

The rationale for using a combination of local therapy (radiotherapy for preservation of the sphincter content) and chemotherapy with CDDP plus IRI in our patient was based on the fact that her complaints had both a prognostically relevant systemic component and a local component.

If metastases are present, they may contain one or both of these histological features [12]. 5-Fluorouracil forms the backbone of the established chemotherapeutic regimen for the treatment of metastatic carcinomas of the rectum, and it tends to be recommended in cases where the adenocarcinoma component is the only constituent detected [13]. If there are metastases exhibiting neuroendocrine differentiation, a regimen of CDDP plus either etoposide or IRI (such as that used in small cell lung cancer [SCLC]) can be used, which is reported to achieve a response rate of 40% [14] and 50% [10], respectively. Theoretically, if the rate of proliferation is low, a higher probability of somatostatin receptor expression can be expected, and treatment with an octreotide analog [15], mammalian target of rapamycin inhibitor [16], sunitinib [17], or lutetium-177 dotatate [18] would be conceivable, analogous to NET treatment. In view of our patient’s high proliferation index, we decided to use a combination of CDDP and IRI. This regimen was also selected with the secondary goal of radiosensitivity in mind. Here, the goal of concurrent chemoradiation was to quickly alleviate problems at the site of the most severe complaints.

Recommendations for local therapy of localized MANEC range from simple local excision analogous to the procedure for early rectal carcinoma to oncological rectal resection [4, 13]. The ENETS guidelines for gastroenteropancreatic NETs also allow for combination treatments [19].

In the case of metastatic NEC, however, the need for surgery is relativized by the fact that even those patients with locally limited disease have lower chances of survival than those with rectal adenocarcinomas. Wang *et al.* [20], for example, found that only 50% of patients with localized MANECs survived 2 years after surgical resection due to the rapid onset of distant metastasis. Definitive chemoradiotherapy has not yet been discussed as an alternative form of treatment or, if at all, only as neoadjuvant chemotherapy. Tanaka *et al*. [13], for example, described the case of a 54-year-old patient with MANEC (female) who developed tumor regression after neoadjuvant chemoradiotherapy followed by surgical resection. Our patient developed a complete response to chemoradiotherapy, which made it possible to dispense with surgical resection and, thus, to preserve the rectum, including the anal sphincter muscle. This outcome had not been reported previously for MANEC but cannot be regarded as unusual in light of the known curative effect of chemoradiotherapy in SCLC. In agreement with this hypothesis, in 2017, Voong *et al*. reported ten cases of pure NEC of the anus and rectum treated with chemoradiation. Locoregional control was achieved for the majority of patients for their remaining lifetime, but all developed distant metastases [21].

Two unexpected findings in our patient were the long-term remission of liver metastases in response to chemotherapy alone and the fact that no further metastases developed. The progression of a single, known lesion made it necessary to administer local therapy.

In view of the location of the primary tumor, its good response to chemoradiotherapy, the limited prospects of complete therapy, and the unfavorable location of the metastases, we opted to perform stereotactic body radiation therapy instead of surgical resection or radiofrequency ablation. This therapeutic approach resulted in local control of metastases without impairment of liver function [22, 23]. The advantages of this approach are obvious, and equivalent methods have not been reported to date.

## Conclusions

This case demonstrates that patients with MANEC can have a good outcome. Our results suggest that even in patients with MANEC with a poorly differentiated neuroendocrine component and metastases, it is reasonable to attempt a local therapy regimen analogous to that used in rectal cancer as an approach to achieve primary tumor control, provided the number of distant metastases is small.

## Abbreviations

CDDP: Cisplatin
CRT: Concomitant chemoradiotherapy
CT: Computed tomography
CTC 5.0: Common toxicity Criteria for Adverse Events version 5.0
ENETS: European Neuroendocrine Tumor Society
IRI: Irinotecan
MANEC: Mixed adenoneuroendocrine carcinoma
NEC: Neuroendocrine carcinoma
SBRT: Stereotactic body radiotherapy
SCLC: Small cell lung cancer
